# Oral cancer prognosis based on clinicopathologic and genomic markers using a hybrid of feature selection and machine learning methods

**DOI:** 10.1186/1471-2105-14-170

**Published:** 2013-05-31

**Authors:** Siow-Wee Chang, Sameem Abdul-Kareem, Amir Feisal Merican, Rosnah Binti Zain

**Affiliations:** 1 Bioinformatics and Computational Biology, Institute of Biological Science, Faculty of Science, University of Malaya, Kuala Lumpur, Malaysia; 2 Department of Artificial Intelligence, Faculty of Computer Science and Information Technology, University of Malaya, Kuala Lumpur, Malaysia; 3 Department of Oral Pathology and Oral Medicine and Periodontology, Oral Cancer Research and Coordinating Centre (OCRCC), Faculty of Dentistry, University of Malaya, Kuala Lumpur, Malaysia

**Keywords:** Oral cancer prognosis, Clinicopathologic, Genomic, Feature selection, Machine learning

## Abstract

**Background:**

Machine learning techniques are becoming useful as an alternative approach to conventional medical diagnosis or prognosis as they are good for handling noisy and incomplete data, and significant results can be attained despite a small sample size. Traditionally, clinicians make prognostic decisions based on clinicopathologic markers. However, it is not easy for the most skilful clinician to come out with an accurate prognosis by using these markers alone. Thus, there is a need to use genomic markers to improve the accuracy of prognosis. The main aim of this research is to apply a hybrid of feature selection and machine learning methods in oral cancer prognosis based on the parameters of the correlation of clinicopathologic and genomic markers.

**Results:**

In the first stage of this research, five feature selection methods have been proposed and experimented on the oral cancer prognosis dataset. In the second stage, the model with the features selected from each feature selection methods are tested on the proposed classifiers. Four types of classifiers are chosen; these are namely, ANFIS, artificial neural network, support vector machine and logistic regression. A *k*-fold cross-validation is implemented on all types of classifiers due to the small sample size. The hybrid model of ReliefF-GA-ANFIS with 3-input features of *drink, invasion* and *p63* achieved the best accuracy (accuracy = 93.81%; AUC = 0.90) for the oral cancer prognosis.

**Conclusions:**

The results revealed that the prognosis is superior with the presence of both clinicopathologic and genomic markers. The selected features can be investigated further to validate the potential of becoming as significant prognostic signature in the oral cancer studies.

## Background

Various machine learning methods have been applied in the diagnosis or prognosis of cancer research, such as, artificial neural networks, fuzzy logic, genetic algorithm, support vector machine and other hybrid techniques [1,2]. From the medical perspective, diagnosis is to identify a disease by its signs and symptoms while prognosis is to predict the outcome of the disease and the status of the patient, whether the patient will survive or recover from the disease or vice versa. In some studies, the researchers have proven that machine learning methods could generate more accurate diagnosis or prognosis as compared to traditional statistical methods [2].

Usually, clinicopathologic data or genomic data are used in researches either involving diagnosis or that with respect to prognosis. Currently, there are some researches that have shown that prognosis results are more accurate when using both clinicopathologic and genomic data. Examples of these are the works in [3] in diffuse large B-cell lymphoma (DLBCL) cancer, the works in [4,5] in breast cancer, [6-10] in oral cancer, and [11] in bladder cancer. However, the number of published articles on researches that combine both clinicopathologic and genomic data are few as compared to that using only clinicopathologic data [2]. In the oral cancer domain, [6] used machine learning techniques in the oral cancer susceptibility studies. They proposed a hybrid adaptive system inspired from learning classifier system, decision trees and statistical hypothesis testing. The dataset includes both demographic data and 11 types of genes. Their results showed that the proposed algorithm outperformed the other algorithms of Naive Bayes, C4.5, neural network and XCS (Evolution of Holland’s Learning Classifier). However, they did not validate their results against the traditional statistical methods. [7] focused on the 5-year overall survival in a group of oral squamous cell carcinoma (OSCC) patients and investigated the effects of demographic data, clinical data and genomic data, and human papillomavirus on the prognostic outcome. They used the statistical methods for the prediction and their results showed that the 5-year overall survival was 28.6% and highlighted the influence of *p53* immunoexpression, age and anatomic localization on OCSS prognosis. However, in this research, no machine learning methods were used and compared. Another oral cancer research that was done by [8,9] was in the oral cancer reoccurrence. Bayesian network was used and compared with ANN, SVM, decision tree, and random forests. They used multitude of heterogeneous data which included clinical, imaging, tissue and blood genomic data. They built a separate classifier for different types of data and combined the best performing classification schemes. They claimed that they had achieved an accuracy of 100% with the combination of all types of data and proved that the prediction accuracy was the best when using all types of data. However, more than 70 markers were required for their final combined classifier.

For the genomic domain, [12] showed that *p63* over expression associates with poor prognosis in oral cancer. Their study showed that cases with diffuse *p63* expression were more aggressive and poorly differentiated and related to a poorer prognosis, these findings supporting the use of *p63* as an additional marker for diagnostic use in oral SCC. In [13], immunohistochemical analysis of protein expression for *p53*, *p63* and *p73* was performed for 40 samples of well-differentiated human buccal squamous-cell carcinomas, with 10 specimens of normal buccal mucosa employed as controls. Their results indicated that both *p73* and *p63* may be involved in the development of human buccal squamous-cell carcinoma, perhaps in concert with *p53*. Similar results were obtained by [14], they have showed that in head and neck squamous carcinomas (HNSC), *p63* was the most frequently expressed (94.7%), followed by *p73* (68.4%) and *p53* (52.6%). Their study indicated that *p63* and *p73* expression may represent an early event in HNSC tumorigenesis and *p73* and *p63* may function as oncogenes in the development of these tumors.

In this research, an oral cancer prognostic model is developed. This research used real-world oral cancer dataset which is collected locally at the Oral Cancer Research and Coordinating Centre (OCRCC), Faculty of Dentistry, University of Malaya, Malaysia. The model takes both clinicopathologic and genomic data in order to investigate the impact of each marker or combination of markers to the accuracy of the prognosis of oral cancer. Five feature selection methods are proposed with the objectives to reduce the number of input variables to avoid over-fitting and to find out an optimum feature subset for oral cancer prognosis. This is followed by the classification procedures which are used to classify the status of the patient after 1–3 years of diagnosis (alive or dead). Four classification methods, from both machine learning and statistical methods, are tested and compared. The objective of this research is to prove that the prognosis is better by using both types of clinicopathologic and genomic markers, and to identify the key markers for oral cancer prognosis using the hybrid of feature selection and machine learning methods.

## Methods

**Figure 1:**
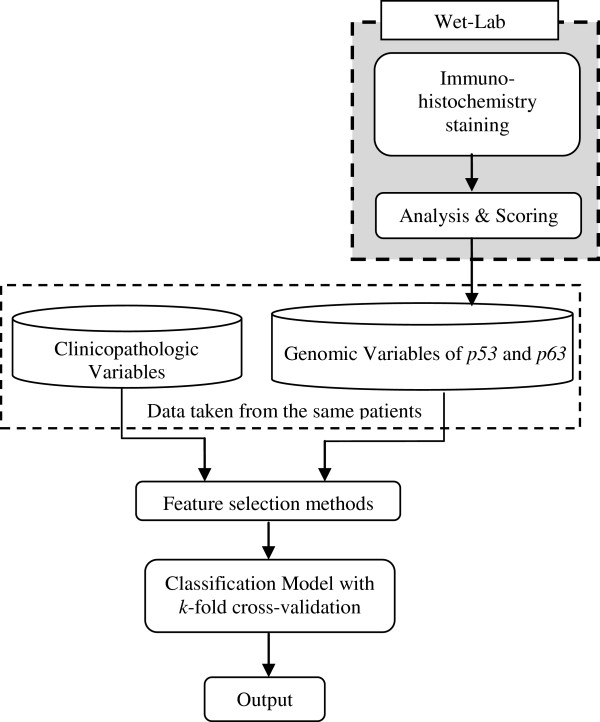
Framework for oral cancer prognostic model.

The framework for the oral cancer prognostic model is shown in Figure 1. Clinicopathologic variables from the OCRCC database and genomic variables from Immunohistochemistry (IHC) staining are fed into the model. Basically, there are three main parts for the oral cancer prognostic model, which are wet-laboratory testing for genomic variables, feature selection methods and the classification models. This research was approved by Medical Ethics Committee, Faculty of Dentistry, University of Malaya.

### Clinicopathologic data

A total of 31 oral cancer cases were selected from the Malaysian Oral Cancer Database and Tissue Bank System (MOCDTBS) coordinated by the Oral Cancer Research and Coordinating Centre (OCRCC), Faculty of Dentistry, University of Malaya. The selection was based on the completeness of the clinicopathologic data, the availability of tissues and the availability of data (some data were not available for use due to medical confidentiality problems).

The selected cases were based on the oral cancer cases seen in the Faculty of Dentistry, University of Malaya and Hospital Tunku Ampuan Rahimah, Klang, a Malaysian government hospital, from the year 2003 to 2007. These cases were diagnosed and followed up and the data were recorded in the standardised forms prepared by the MOCDTBS. Later, MOCDTBS transcribed all the data from paper to an electronic version and stored in the database. All the cases selected were diagnosed as squamous cell carcinomas (SCC). Table 1 shows the 1 to 3-year survival for these 31 cases.

**Table 1:** 1-year, 2-year and 3-year survival

**Duration of follow-up**	**Survival**	**No**	**%**
1-year	Survive	27	87.1
	Dead	4	12.9
	Lost of follow-up	0	0.0
2-year	Survive	19	61.3
	Dead	10	32.3
	Lost of follow-up	2	6.5
3-year	Survive	17	54.8
	Dead	11	38.7
	Lost of follow-up	3	9.7

Basically, three types of data are available for each oral cancer case, namely, social demographic data (risk factors, ethnicity, age, occupation, marital status and others), clinical data (type of lesion, size of lesion, primary site, clinical neck node and etc.), and pathological data (pathological TNM, neck node metastasis, bone invasion, tumour thickness and etc.). Pathological data were obtained from the biopsy reports before and after surgical procedures. In this research, we referred to the clinical and pathological data as clinicopathologic data. Based on the discussions with two oral cancer clinicians, Prof. Rosnah Binti Zain and Dr Thomas George Kallarakkal, 15 key variables had been identified as important prognostic factors of oral cancer. The selected clinicopathologic variables are listed in Table 2(a).

**Table 2:** Description and membership function for clinicopathologic and genomic variables

**(a) Clinicopathologic variables**		
**Name**	**Description**	**Name & parameters of membership function**
Age	Age at diagnosis	1 - 40–50, 2 - >50-60, 3 - >60-70, 4 - >70
Eth	Ethnicity	1 - Malay, 2 - Chinese, 3 - Indian
Gen	Gender	1 - Male, 2 - Female
Smoke	Smoking habit	1 - Yes, 2 - No
Drink	Alcohol drinking habit	1 - Yes, 2 - No
Chew	Quid chewing habit	1 - Yes, 2 - No
Site	Primary site of tumor	1 - Buccal mucosa, 2 - tongue
		3 - floor, 4 - others
Subtype	Subtype and differentiation for SCC	1 - Well differentiated
		2 - moderate differentiated
		3 - poorly differentiated
Inv	Depth of Invasion front	1 - Non-cohesive, 2 - cohesive
Node	Neck nodes	1 - Negative, 2 - positive
PT	Pathological tumor staging	1 - T1, 2 - T2, 3 - T3, 4 - T4
PN	Pathological lymph nodes	1 - N0, 2- N1, 3- N2A, 4- N2B
Stage	Overall stage	1 - I, 2 - II, 3 - III, 4 - IV
Size	Size of tumor	1 - 0-2 cm, 2 - >2-4 cm, 3 - >4-6 cm, 4 - >6 cm
Treat	Type of treatment	1 - Surgery only
		2 - Surgery + Radiotherapy
		3 - Surgery + Chemotherapy
**(b) Genomic variables**		
**Name**	**Description**	**Name and parameters of membership function**
*p53*	Tumor suppressor gene	1 - negative, 2 - positive
*p63*	Tumor suppressor gene	1 - negative, 2 - positive

### Genomic data

Two genomic variables had been identified through the literature studies and discussions with oral pathologists, from the Department of Oral Pathology and Oral Medicine and Periodontology, Faculty of Dentistry, University of Malaya. Both of these variables are tumour suppressor genes, namely, *p53* and *p63. p53* is the most frequently associated marker in the head and neck cancers [7,15]. *p53* is called the “Guardian of the genome”, it is important in maintaining genomic stability, progression of cell cycle, cellular differentiation, DNA repair and apoptosis. It is difficult to demonstrate *p53* protein in normal tissues using immunohistochemistry procedures due to its high catabolic rate; however mutated *p53* exhibits a much lower catabolic rate and accumulates in the cells [15]. In addition, *p63* gene, a homolog of the *p53* is located in chromosome *3q21-29*, and its amplification has been associated with prognostic outcome in oral cancer [11,16]. The *p63* gene is highly expressed in the basal or progenitor layers of many epithelial tissues.

The cases selected were the same as in the clinicopathologic data. Immunohistochemistry (IHC) staining was performed on the selected formalin-fixed paraffin embedded oral cancer tissues to obtain the results for the selected genomic variables. The archival formalin-fixed paraffin embedded tissues were obtained from the Oral Pathology Diagnostic Laboratory, Faculty of Dentistry, University of Malaya. The tissues containing the tumour were cored and re-embedded and made into Tissue Macroarray blocks (TMaA). A total of 4-*μ*m-thick sections of the resulting TMaA blocks were cut and placed on the poly-L-lysine-coated glass slides for IHC staining. The samples were mounted on the glass slides and ready for IHC staining. In this research, the Dako REAL™ EnVision™ Detection Kit was used. In total, 15 TMaA slides with 31 oral cancer cases were stained. Two types of antibodies were used namely Monoclonal Mouse Anti-Human *p53* protein, clone *318-6-11* for *p53* and Monoclonal Mouse Anti-Human *p63* protein, clone *4A4* for *p63*.

**Figure 2:**
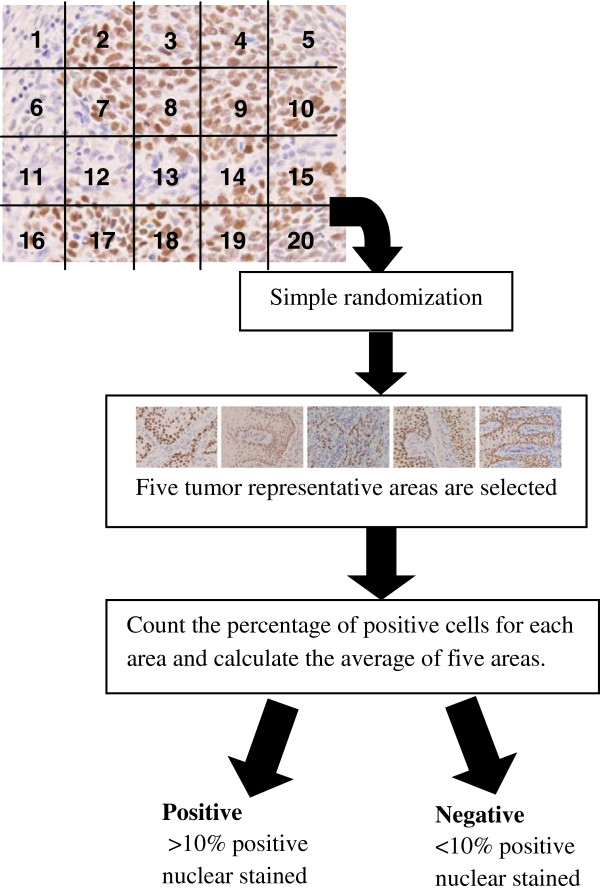
Procedures for IHC results analysis and scoring.

The results of the staining were analyzed and the images were captured by using an image analyzer system which included Nikon Eclipse E400 Microscope with CFI plan Fluor 40X objective for measurements, QImaging Evolution digital colour cooled camera with 5.0 megapixels, a personal computer (Pentium 4, 2.5Ghz, 2GB RAM) and MediaCybernatics Image Pro Plus version 6.3 image analysis software. Each slide was first examined under the microscope with lower objective, that is, the 4X objective. Cases were considered sufficient for evaluation if there were tumour cells presented in the sections. Next, the slide was divided into 20 grid cells and numbered accordingly from left to right. A simple randomization program was used to generate random numbers. For each case, five tumour representative areas were selected. If the number falls on the non-tumour representative area, the next number (cell) was chosen until all five areas were selected. Next, the five selected areas were examined under the microscope using a higher objective, that is, the 40X objective and the images were captured. The percentage of the positive nuclear cells for each area was counted and the average for five areas was calculated. The staining result is considered positive if there is more than 10% positive nuclear stained, in accordance with the practice used in the previous studies [7,17]. Figure 2 shows the flowchart for the IHC results analysis and scoring process. The results obtained from the IHC staining are combined with the clinicopathologic variables and served as the inputs for the feature selection module. The combined dataset is further divided into two groups, which are Group 1 with clinicopathologic variables (15 variables) only and Group 2 with both of the clinicopathologic and genomic variables (17 variables). We need to emphasize that the genomic variables were obtained from the same corresponding cases from which the clinicopathologic variables (Group 1) were obtained. Thus, if the clinicopathologic variables were that of Case 1, then, the genomic variables were from the same case.

### Feature selection

In this research, the purpose of feature selection is to find an optimal number of features for the small sample of oral cancer prognosis data. Five feature selection methods have been selected and compared, which are, Pearson’s correlation coefficient (CC) [18], and Relief-F [19] as the filter approach, genetic algorithm (GA) [20,21] as the wrapper approach, CC-GA and ReliefF-GA as the hybrid approach.

### Genetic algorithm (GA)

In the feature subset selection problem, a solution is a specific feature subset that can be encoded as a string of binary digits (bits). Each feature is represented by binary digits of *0* or *1*. For example, in the oral cancer prognosis dataset, if the solution is a *011001000010000* string of 15 binary digits, it indicates that features 2, 3, 6, and 11 are selected as the feature subset [21]. The initial population is generated randomly to select a subset of variables. In this proposed GA feature selection method, if the features are all different, the subset is included in the initial population. If not, it is regenerated until an initial population with the desired size for the feature subset (*n*-input model) is created.

**Figure 3:**
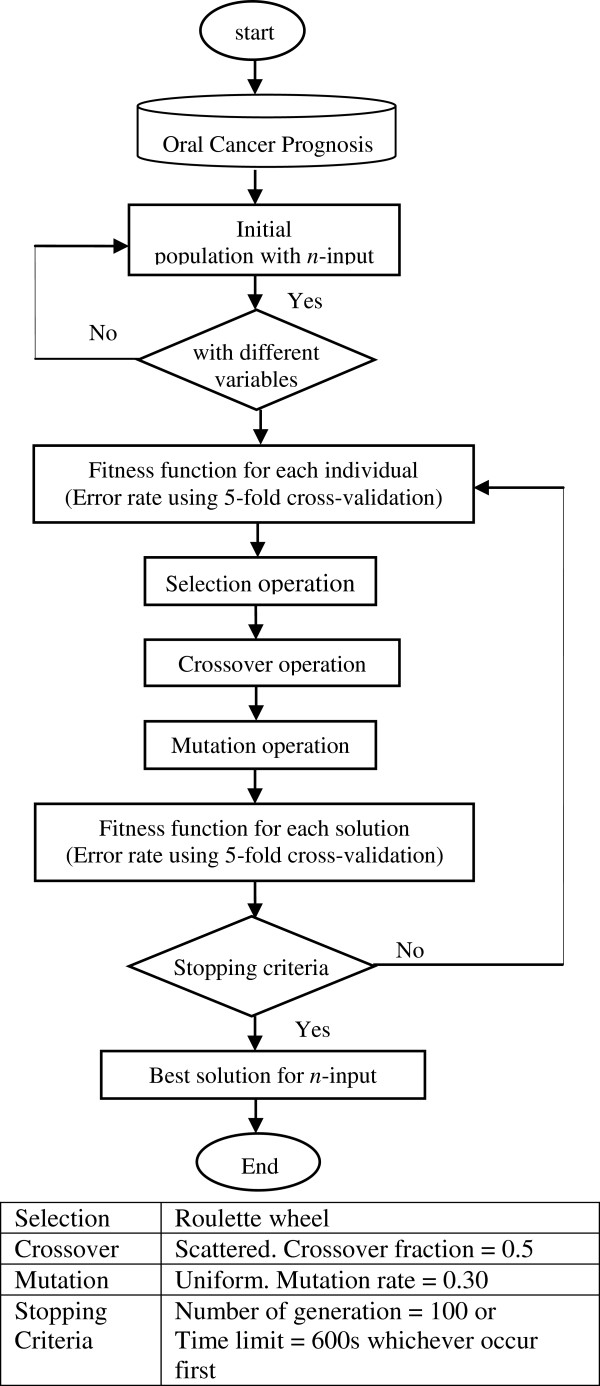
Genetic algorithm feature selection flowchart.

The fitness function used in this method is a classifier to discriminate between two groups, which are alive and dead after 3-year of diagnosis. The mean square error rate of the classification is calculated using a 5-fold cross-validation. The fitness function is the final mean square error rate obtained. The subset of variables with the lowest error rate is selected. Figure 3 shows the flowchart and the criteria used for the GA feature selection approach.

### Pearson’s correlation coefficient (CC)

Pearson’s correlation coefficient, *r*, is use to see if the values of two variables are associated. In this research, *r* is calculated and ranked for each of the feature input and the one with the highest *r* is selected. For example, for the 3-input model, the top three inputs with the highest *r* value are selected. This is repeated for the *n*-input models for both Group 1 and Group 2.

### Relief-F

Relief-F is the extension to the original Relief algorithm, which is able to deal with noisy and incomplete datasets as well as multi-class problems. The key idea of Relief is to estimate attributes according to how well their values distinguish among instances that are near to each other [18]. In this research, each feature input is ranked and weighted using the *k*-nearest neighbours classification, in which *k* = 1. The top features with large positive weights are selected for both groups of dataset.

### Pearson’s correlation coefficient and genetic algorithm (CC-GA)

This is the hybrid feature selection approach which consists of two stages: first, it is a filter approach which calculates the correlation coefficient, *r*, and second, it is a wrapper approach of GA. In the first stage, 10 features with the highest *r* are selected and fed into the second stage of the GA approach. The procedures of the GA are the same as that described in the previous section.

### Relief-F and genetic algorithm (ReliefF-GA)

This hybrid feature selection approach consists of two stages: first, it is the filter approach of Relief-F, and second, it is a wrapper approach of GA. In the first stage, 10 features with the highest weights are selected and fed into the second stage of the GA approach. In the second stage, *n*-input is selected for both Group 1 and Group 2.

### Selection of *n*-input models

Before the implementation of the feature selection method, a simple GA was run to find out the optimal number of inputs (*n-input model*) from the 17 inputs of clinicopathologic and genomic data. The number of inputs with lower mean square error rate was chosen. The error rate for each *n*-input model is shown in Table 3, which shows that for Group 1, there are four models with the lowest error rate of 0.3871, which are the 3-input, 4-input, 5-input, and 6-input model. Meanwhile, for Group 2, the model with the lowest error rate is the 3-input model with an error rate of 0.2581. In this case, for comparison purposes, the number of inputs between 3-input to 7-input are chosen. Hence *n* is set as *n* = 3, 4, 5, 6, 7 for the feature selection methods.

**Table 3:** **Mean square error rate for *n*-input model**

	**Group 1**	**Group 2**
1-input	0.3881	0.3626
2-input	0.4193	0.2903
3-input	0.3871	0.2581
4-input	0.3871	0.2903
5-input	0.3871	0.3226
6-input	0.3871	0.3548
7-input	0.4571	0.3548
8-input	0.4839	0.4194
9-input	0.5161	0.4516

### Classification

Next, the data with *n* selected features are fed into the classification models. The final output is the classification accuracy for oral cancer prognosis, which classifies the patients as alive or dead after subsequent years of diagnosis with the optimum feature of subset. Four classification methods were experimented with and their results were subsequently compared, these are ANFIS, artificial neural network (ANN), support vector machine and logistic regression.

In order to obtain accurate estimate results, cross-validation (CV) was used. CV provides an unbiased estimation, however CV presents high variance with small samples in some studies [22]. In this research, a *5*-fold cross-validation was implemented with each of the classifiers. 5-fold cross-validation was chosen over the commonly use 10-fold cross-validation due to the small sample size; hence, it will leave more instances for validation and has lower variance [23]. In 5-fold cross-validation, the 31 samples of oral cancer prognosis data were divided into 5 subsets of equal size and trained for 5 times, each time leaving out a sample as validation data.

### Adaptive neuro-fuzzy inference system (ANFIS)

ANFIS implements the Takagi-Sugeno fuzzy inference system. The details for ANFIS can be found in [24,25] respectively.

**Figure 4:**
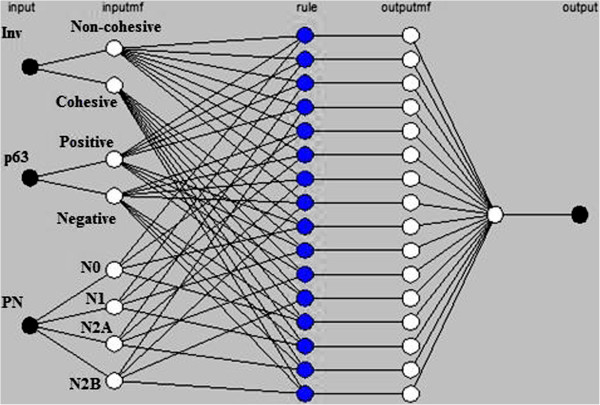
ANFIS rules for a 3-input model.

In the input layer, the number of input is defined by *n,* with *n* = 3, 4, 5, 6, 7. In the input membership (inputmf) layer, the number of membership function is defined by *m*_*i*_, with *i* = 2, 3, 4. The rules generated are based on the number of input and the number of input membership functions, and it is represented as (*m*_*2*_^*n1*^ x *m*_*3*_^*n2*^ x *m*_*4*_^*n3*^*)* rules, in which *n*_*1*_*, n*_*2*_*,* and *n*_*3*_ represent the number of input with *m*_*i*_ membership functions respectively, and *n*_*1*_ *+ n*_*2*_ *+ n*_*3*_ *= n.* For example, in the ANFIS with 3-input, *x, y, and z,* in which input *x* has 2 membership functions, input *y* has 2 membership functions, and input *z* has 4 membership functions, hence the number of rules generated is (*2*^*2*^ × *3*^*0*^× *4*^*1*^) = 16 rules, as shown in Figure 4.

The rules generated are the output membership functions which will be computed as the summation of the contribution from each rule towards the overall output. The output is the survival condition, either alive or dead after 3-year of diagnosis. The output is set as 1 for dead and −1 for alive; the pseudo-code is as below:

if output ≥ 0

then set output = 1, classify as dead

else output < 0,

then set output = −1, classify as alive

The membership functions were obtained according to the categorical variables that has been set through the discussions with two oral cancer clinicians as mentioned in section Clinicopathologic Data. The type of membership function used was the Gaussian and the name and parameters of membership functions for each input variable are shown in Table 2(a) for the clinicopathologic variables and 2(b) for the genomic variables. Each ANFIS was run for 5 epochs for the optimum result.

### Artificial neural network (ANN)

The ANN employed in this research is the multi-layered feed forward (FF) neural network, which is the most common type of ANN [26]. The FF neural network was trained using the Levenberg-Marquardt algorithm. In this research, one hidden layer with five neurons (achieved the best results) was used and FF neural network was run for 5 epochs (achieved the best results). The training stopped when there was no improvement on the mean squared error for the validation set.

### Support vector machine (SVM)

For the purpose of this research, a widely used SVM tool which is LIBSVM [27] was used. There are 2 steps involved in the LIBSVM; (1) the dataset was trained to obtain a model and (2) the model was used to predict the information for the testing dataset. The details for LIBSVM can be found in [27,28] respectively. Linear kernel was used in this research.

### Logistic regression (LR)

Logistic regression (LR) was selected as the benchmark test for the statistical method. LR is the most commonly used statistical method for the prediction of diagnosis and prognosis in medical research. LR is the prediction of a relationship between the response variable *y* and the input variables *x*_*i*_[29]. In this research, multiple logistic regression is used.

### Experiment

The oral cancer dataset with 3-year prognosis is used in this experiment. First, the oral cancer prognosis dataset was divided into two groups; Group 1 consisted of clinicopathologic variables only (15 variables) and Group 2 consisted of clinicopathologic and genomic variables (17 variables). Next, feature selection methods were implemented on both groups to select the key features for the *n*-input model. Lastly, the classifiers with 5-fold cross-validation were tested on the *n*-input model. The results obtained from the 5-fold cross-validation were averaged in order to produce the overall performance of the algorithm. The measures used to compare the performance of the proposed methods were sensitivity, specificity, accuracy and area under the Receiver Operating Characteristic (ROC) curve (AUC).

## Results

### Group 1 (clinicopathologic variables)

Table 4 shows the features selected for the proposed feature selection methods for Group 1. Next, the *n*-input models generated from each feature selection methods were tested with the proposed classification methods. Table 5 shows the classification results for ANFIS, ANN, SVM and LR.

**Table 4:** Feature subset selected for group 1

**Method**	**Feature subset selected**
GA	
3-input	*Gen,Smo,PN*
4-input	*Dri,Inv,PN,Size*
5-input	*Dri,Node,PT,PN,Size*
6-input	*Age,Gen,Smo,Inv,PT,Size*
7-input	*Age,Eth,Chew,Inv,Node,PN,Size*
CC	
3-input	*Age,Inv,PN*
4-input	*Age,Gen,Inv,PN*
5-input	*Age,Gen,Inv,PN,Size*
6-input	*Age,Gen,Inv,PN,Sta,Size*
7-input	*Age,Gen,Dri,Inv,PN,Sta,Size*
ReliefF	
3-input	*Eth,Dri,Sta*
4-input	*Age,Eth,Dri,Sta*
5-input	*Age,Eth,Dri,Sta,Tre*
6-input	*Age,Eth,Gen,Dri,Sta,Tre*
7-input	*Age,Eth,Gen,Dri,PT,Sta,Tre*
CC-GA	
3-input	*PT,PN,Sta*
4-input	*Dri,Inv,PN,Size*
5-input	*Age,Gen,Inv,PN,Size*
6-input	*Gen,Dri,Node,PT,PN,Sta*
7-input	*Gen,Dri,Chew,Inv,Node,PN,Size*
ReliefF-GA	
3-input	*Gen,Inv,Node*
4-input	*Gen,Dri,Inv,Node*
5-input	*Gen,Dri,Inv,Node,PT*
6-input	*Eth,Gen,Dri,Inv,Node,PT*
7-input	*Age,Eth,Gen,Smo,Dri,Node,Tre*

**Table 5:** Classification accuracy and AUC for group 1

**Feature selection**	**3-input**		**4-input**		**5-input**		**6-input**		**7-input**	
	**%**	**AUC**	**%**	**AUC**	**%**	**AUC**	**%**	**AUC**	**%**	**AUC**
ANFIS										
GA	70.95	0.66	67.42	0.61	64.76	0.63	58.57	0.55	57.62	0.54
CC	58.10	0.53	74.76	0.70	51.43	0.43	57.62	0.50	64.29	0.58
ReliefF	61.43	0.53	50.59	0.50	58.10	0.50	64.29	0.54	64.29	0.54
CC-GA	44.76	0.44	67.62	0.57	63.81	0.55	64.29	0.54	57.62	0.52
ReliefF-GA	67.14	0.55	60.48	0.59	67.62	0.59	51.90	0.47	64.76	0.57
ANN										
GA	45.52	0.53	52.43	0.53	45.05	0.47	48.38	0.52	45.33	0.50
CC	54.48	0.61	53.57	0.59	51.29	0.58	51.29	0.51	52.33	0.53
ReliefF	51.52	0.48	41.62	0.47	46.05	0.49	46.05	0.48	44.10	0.48
CC-GA	49.24	0.51	49.48	0.52	46.67	0.49	48.29	0.49	50.48	0.51
ReliefF-GA	50.24	0.55	52.86	0.59	56.76	0.58	47.00	0.51	50.05	0.54
SVM										
GA	60.95	0.53	61.43	0.51	58.10	0.48	58.10	0.46	61.43	0.49
CC	60.95	0.53	60.95	0.53	58.10	0.46	51.43	0.41	51.43	0.41
ReliefF	54.29	0.44	50.95	0.42	51.43	0.42	48.10	0.40	50.95	0.45
CC-GA	63.81	0.55	61.43	0.51	58.10	0.46	58.10	0.48	58.10	0.49
ReliefF-GA	64.29	0.50	64.29	0.50	64.29	0.50	64.29	0.50	54.76	0.46
LR										
GA	64.29	0.56	67.62	0.60	64.76	0.55	68.10	0.64	64.29	0.60
CC	64.29	0.56	60.48	0.57	67.62	0.61	67.62	0.61	64.29	0.58
ReliefF	50.59	0.44	50.59	0.44	48.10	0.39	41.43	0.34	44.29	0.39
CC-GA	67.62	0.57	67.62	0.60	61.43	0.51	70.95	0.72	64.76	0.67
ReliefF-GA	54.29	0.54	51.43	0.52	61.43	0.62	47.62	0.55	48.10	0.51

From Table 5, it can be seen that ANFIS with the CC-4-input model, obtained the best accuracy of 74.76% and an AUC of 0.70. For the ANN results, the model with the highest accuracy is the ReliefF-GA-5-input model with an accuracy of 56.76% and an AUC of 0.58. Whereas, for the SVM classifier, the models with the best accuracy are ReliefF-GA-3-input to 6-input models with an accuracy of 64.29% and an AUC of 0.50. As for LR classification, the best model is the CC-GA-6-input model with an accuracy of 70.95% and an AUC of 0.72. The results obtained from both ANN and SVM showed low accuracy (56.76% & 64.29% respectively) and low AUC (0.58 and 0.50 respectively), hence, indicated that these two are not the suitable classifiers to use for Group 1.

### Group 2 (clinicopathologic and genomic variables)

The same experiments were carried out on Group 2, which is the combination of clinicopathologic and genomic variables. The selected features for each n-input model are listed in Table 6. Table 6 shows that almost all the feature selection methods included the genomic variable as one of the key features, except for the ReliefF-3-input and ReliefF-4-input.

**Table 6:** Feature subset selected for group 2

**Method**	**Feature subset selected**
GA	
3-input	*Inv,Node,p63*
4-input	*Gen,Inv,Size,p53*
5-input	*Age,PT,PN,Size,p53*
6-input	*Age,PT,PN,Size,Tre,p53*
7-input	*Age,Eth,Smo,PT,PN,Size,p53*
CC	
3-input	*Inv,PN,p63*
4-input	*Age,Inv,PN,p63*
5-input	*Age,Gen,Inv,PN,p63*
6-input	*Age,Gen,Inv,PN,Size,p63*
7-input	*Age,Gen,Inv,PN,Size,p53,p63*
ReliefF	
3-input	*Age,Eth,Dri*
4-input	*Age,Eth,Dri,Tre*
5-input	*Age,Eth,Dri,Tre,p53*
6-input	*Age,Eth,Dri,Tre,p53,p63*
7-input	*Age,Eth,Gen,Dri,Tre,p53,p63*
CC-GA	
3-input	*Inv,Node,p63*
4-input	*Gen,Inv,Size,p53*
5-input	*Age,Dri,PN,Size,p53*
6-input	*Gen,Inv,Node,PN,Size,p53*
7-input	*Gen,Dri,Inv,Node,PN,Size,p53*
ReliefF-GA	
3-input	*Dri,Inv,p63*
4-input	*Dri,Inv,Tre,p63*
5-input	*Age,Gen,Smo,Dri,p63*
6-input	*Age,Gen,Smo,Dri,Inv,p63*
7-input	*Age,Eth,Inv,Sta,Tre,p53,p63*

For Group 2 using the ANFIS classification (Table 7), there are five models with an accuracy of above 70%, these are namely, GA-3-input, CC-GA-3-input, CC-GA-4-input, ReliefF-GA-3-input and ReliefF-GA-4-input. The best results were obtained from the ReliefF-GA-3-input and ReliefF-GA-4-input with an accuracy of 93.81% and an AUC of 0.90 and the features selected for the ReliefF-GA-3-input are *drink*, *invasion,* and *p63* while the features selected for the ReliefF-GA-4-input are *drink*, *invasion, treatment* and *p63* (refer Table 6).

**Table 7:** Classification accuracy and AUC for group 2

**Feature selection**	**3-input**		**4-input**		**5-input**		**6-input**		**7-input**	
	**%**	**AUC**	**%**	**AUC**	**%**	**AUC**	**%**	**AUC**	**%**	**AUC**
ANFIS										
GA	74.76	0.74	67.62	0.70	41.90	0.40	58.57	0.58	35.71	0.36
CC	58.10	0.48	58.10	0.52	51.90	0.48	48.57	0.46	61.90	0.59
ReliefF	54.29	0.47	44.29	0.38	48.10	0.53	67.14	0.62	67.14	0.62
CC-GA	74.76	0.70	70.48	0.71	54.76	0.57	61.43	0.61	64.29	0.65
ReliefF-GA	93.81	0.90	93.81	0.90	65.71	0.63	64.76	0.62	68.10	0.67
ANN										
GA	45.14	0.50	51.48	0.55	45.81	0.49	46.14	0.50	47.71	0.51
CC	46.24	0.46	49.38	0.49	46.14	0.50	57.38	0.58	55.48	0.57
ReliefF	40.62	0.48	43.24	0.49	47.71	0.50	49.48	0.51	48.76	0.50
CC-GA	49.38	0.52	53.90	0.60	47.05	0.52	44.76	0.48	55.19	0.57
ReliefF-GA	84.62	0.83	73.38	0.75	48.00	0.52	51.57	0.53	45.86	0.47
SVM										
GA	74.76	0.70	54.76	0.51	70.95	0.65	60.95	0.55	50.95	0.42
CC	64.76	0.55	64.76	0.55	64.76	0.55	67.62	0.56	67.62	0.62
ReliefF	54.29	0.44	54.29	0.44	44.29	0.36	48.10	0.46	34.76	0.28
CC-GA	74.76	0.70	54.76	0.51	61.43	0.50	58.10	0.54	61.43	0.57
ReliefF-GA	74.76	0.70	71.43	0.68	74.76	0.70	74.43	0.66	54.76	0.53
LR										
GA	74.76	0.70	63.81	0.64	67.14	0.57	54.76	0.43	54.29	0.47
CC	71.43	0.67	71.43	0.67	61.43	0.59	68.10	0.65	61.43	0.59
ReliefF	50.59	0.45	48.10	0.39	48.10	0.41	44.76	0.43	41.43	0.41
CC-GA	74.76	0.70	63.81	0.64	60.48	0.61	64.29	0.63	60.48	0.54
ReliefF-GA	74.76	0.70	74.76	0.70	71.43	0.68	58.10	0.55	61.43	0.60

As shown in Table 7, the FF neural network together with the ReliefF-GA-3-input model achieved the best result with an accuracy of 84.62% and an AUC of 0.83. For SVM classification, the classification results are generally better in Group 2 when compared to Group 1 (Table 5) with some exceptions (GA-3-input, GA-7-input, CC-GA-4-input, ReliefF-5-input and ReliefF-7-input). The best accuracy in Group 2 is obtained by the GA-3-input, CC-GA-3-input, ReliefF-GA-3-input, and ReliefF-GA-5-input with an accuracy of 74.76% and an AUC of 0.70. Whereas, for LR classification in Group 2, GA-3-input, CC-GA-3-input, ReliefF-GA-3-input and ReliefF-GA-4-input achieved the best classification accuracy of 74.76% and an AUC of 0.70.

### Comparison of the results for group 1 and group 2

**Figure 5:**
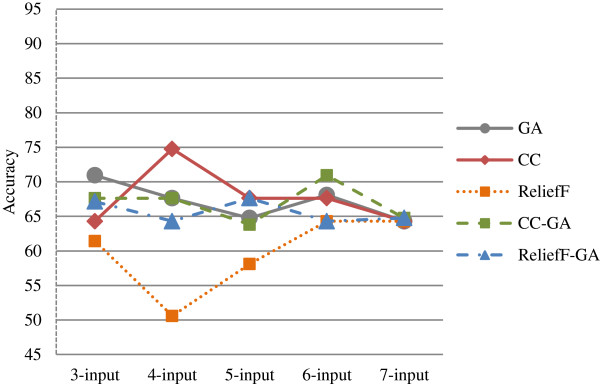
Graphs for best accuracy for n-input model based on feature selection method for Group 1.

**Figure 6:**
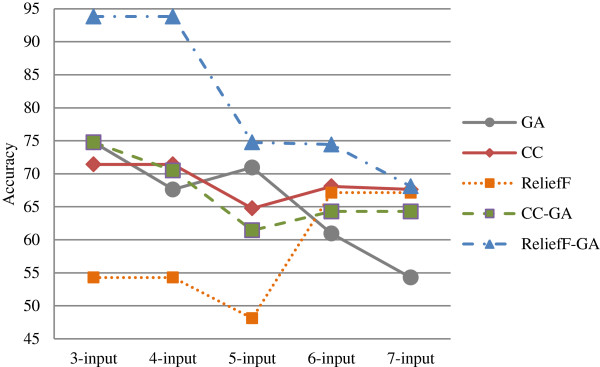
Graphs for best accuracy for n-input model based on feature selection method for Group 2.

This section discusses and compares the results generated from different classification methods for both Group 1 and Group 2. Table 8 summarizes the best accuracy for the *n*-input model based on the feature selection method for Group 1 and Group 2. The summary is also depicted in the graph as shown in Figure 5 and Figure 6 respectively.

**Table 8:** **Best accuracy for *n*-input model based on feature selection method**

**Feature selection method**	**Group 1**					**Group 2**				
	***n-*input model**					***n*-input model**				
	**3**	**4**	**5**	**6**	**7**	**3**	**4**	**5**	**6**	**7**
GA	70.95	67.62	64.76	68.10	64.29	74.76	67.62	70.95	60.95	54.29
CC	64.29	74.76	67.62	67.62	64.29	71.43	71.43	64.76	68.10	67.62
ReliefF	61.43	50.59	58.10	64.29	64.29	54.29	54.29	48.10	67.14	67.14
CC-GA	67.62	67.62	63.81	70.95	64.76	74.76	70.48	61.43	64.29	64.29
ReliefF-GA	67.14	64.29	67.62	64.29	64.76	93.81	93.81	74.76	74.43	68.10

For Group 1 (Figure 5), the correlation coefficient (CC) feature selection method performed better than the other methods with the highest accuracy of 74.76% in the 4-input model. There are three models that achieved accuracies of above 70%; the other two are GA-3-input and CC-GA-6-input. ReliefF feature selection method obtained the worst results when compared to the other methods

As regards to Group 2 (Figure 6), the ReliefF-GA feature selection method outperformed the others in all the *n*-input models, with the highest accuracy of 93.81%. There are ten models with accuracies above 70% as shown in Table 8; this confirms that Group 2 which includes genomic variables achieved higher accuracy with feature selection methods. In addition, most of the models with higher accuracy are the lower input models with 3 or 4-input only.

**Figure 7:**
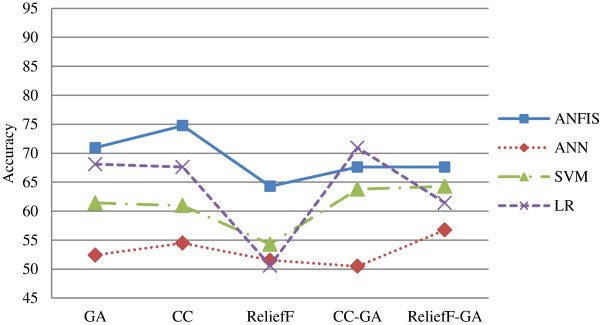
Graphs for best accuracy by classification method for Group 1.

**Figure 8:**
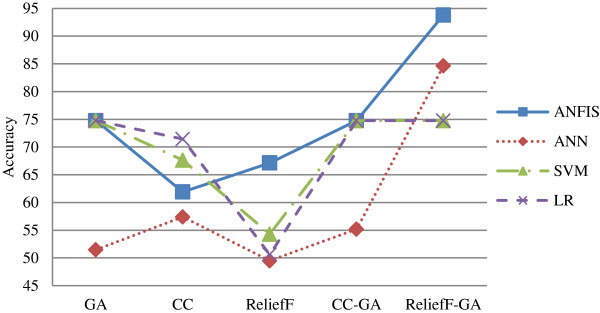
Graphs for best accuracy by classification method for Group 2.

Next, Table 9 lists the best accuracy by classification method and the graphs are depicted in Figures 7 and 8 for both Group 1 and Group 2 respectively.

**Table 9:** Best accuracy by classification method

**Feature selection method**	**Group 1**				**Group 2**			
	**Classification method**				**Classification method**			
	**ANFIS**	**ANN**	**SVM**	**LR**	**ANFIS**	**ANN**	**SVM**	**LR**
GA	70.95	52.43	61.43	68.10	74.76	51.48	74.76	74.76
CC	74.76	54.48	60.95	67.62	61.90	57.38	67.62	71.43
ReliefF	64.29	51.52	54.29	50.59	67.14	49.48	54.29	50.59
CC-GA	67.62	50.48	63.81	70.95	74.76	55.19	74.76	74.76
ReliefF-GA	67.62	56.76	64.29	61.43	93.81	84.62	74.76	74.76

From Figure 7, ANFIS performed the best in Group 1 when compared to other classification methods for all types of feature selection methods except CC-GA method. All the classification methods performed worst in ReliefF feature selection method except for ANN. ANN had the lowest accuracy rate if compared to other methods.

Whereas, in Group 2 as shown in Figure 8, ANFIS outperformed the other classification methods except in CC feature selection method. The best accuracy is achieved by ANFIS in ReliefF-GA method with the accuracy of 93.81% (Table 9). In general, all classification methods performed better in CC-GA and ReliefF-GA hybrid feature selection methods except for SVM and LR. As with Group 1, ANN had the lowest classification rate except in ReliefF-GA method. Overall, the performance of the classification method is better in Group 2 as compared to Group 1. Table 10 summarizes the best models with their selected features for both Group 1 and Group 2. All the models with the accuracy of 70% and above are selected.

**Table 10:** Best models with accuracy, AUC, classification method and selected features

	**Accuracy**	**AUC**	**Classification method**	**Selected features**
Group 1				
CC-3-input	74.76	0.70	ANFIS	*Age,Inv,PN*
GA-3-input	70.95	0.66	ANFIS	*PT,PN,Sta*
CC-GA-6-input	70.95	0.73	LR	*Gen,Dri,Node,PT,PN,Sta*
Group 2				
ReliefF-GA-3-input	93.81	0.90	ANFIS	*Dri,Inv,p63*
ReliefF-GA-4-input	93.81	0.90	ANFIS	*Dri,Inv,Tre,p63*
ReliefF-GA-3-input	84.62	0.83	ANN	*Dri,Inv,p63*
ReliefF-GA-3-input	84.62	0.83	ANN	*Dri,Inv,p63*
GA-3-input	74.76	0.74	ANFIS	*Inv,Node,p63*
CC-GA-3-input	74.76	0.70	ANFIS	*Inv,Node,p63*
CC-GA-3-input	74.76	0.70	SVM	*Inv,Node,p63*
CC-GA-3-input	74.76	0.70	LR	*Inv,Node,p63*
ReliefF-GA-3-input	74.76	0.70	SVM	*Dri,Inv,p63*
ReliefF-GA-3-input	74.76	0.70	LR	*Dri,Inv,p63*
Relief-GA-4-input	74.76	0.70	LR	*Dri,Inv,Tre,p63*
Relief-GA-5-input	74.76	0.70	SVM	*Age,Gen,Smo,Dri,p63*
Relief-GA-6-input	74.43	0.66	SVM	*Age,Gen,Smo,Dri,Inv,p63*
Relief-GA-4-input	73.38	0.75	ANN	*Dri,Inv,Tre,p63*
Relief-GA-4-input	71.43	0.68	SVM	*Dri,Inv,Tre,p63*
Relief-GA-5-input	71.43	0.68	LR	*Age,Gen,Smo,Dri,p63*
CC-3-input	71.43	0.67	LR	*Inv,PN,p63*
CC-4-input	71.43	0.67	LR	*Age,Inv,PN,p63*
CC-GA-4-input	70.48	0.71	ANFIS	*Gen,Inv,Size,p53*

From Table 10, the models with the highest accuracy are ReliefF-GA-3-input and ReliefF-GA-4-input from Group 2 with ANFIS classification, the accuracy is 93.81% and AUC of 0.90. The features selected are *Drink, Invasion* and *p63* and *Drink, Invasion, Treatment,* and *p63* respectively. This is followed by the ReliefF-GA-3-input model from Group 2 with ANN classification, with the accuracy of 84.62% and AUC of 0.83. Most of the best models are generated from the ReliefF-GA feature selection method; this proves that the features selected by this method are the optimum features for the oral cancer prognosis dataset.

## Discussions

The results shown meets the objective of this research, namely, the classification performance is much better with the existence of genomic variables in Group 2. From the results in Table 10, the best feature selection method for oral cancer prognosis is ReliefF-GA with ANFIS classification. This shows that the ANFIS is the most optimum classification tool for oral cancer prognosis.

Since there are two top models with the same accuracy, hence, the simpler one is recommended for further works in similar researches which is the ReliefF-GA-3-input model with ANFIS classification, and the optimum subset of features are *Drink, Invasion* and *p63*. These findings confirmed that of some previous studies. Alcohol consumption has always been considered as a risk factor and one of the reasons for poor prognosis of oral cancer [30-33]. Walker D et al. [34] showed that the depth of invasion is one of the most important predictors of lymph node metastasis in tongue cancer. The different researches done by [35-38], discovered a significant link between the depth of invasion and oral cancer survival. As regards to *p63*, [12-14] showed that p63 over expression associates with poor prognosis in oral cancer.

A comparison between the current methodology and the other methods in the literature was done and shown in Table 11. Nevertheless, direct comparisons cannot be performed since different datasets have been employed in each case. In this comparison, we compared the studies which utilized at least both types of clinical and genomic data in oral cancer. In general, the proposed methodology exhibits superior results compared to the other methods except the work done by [8,9] which claimed to achieve an accuracy of 100%. However, they employed different classifiers for different source of data and more than 70 markers were required for their final combined classifier. A significant advantage of our proposed methodology is only three optimum markers are needed with a single classifier for both types of clinicopathologic and genomic data to obtain high accuracy result. It is hope that the proposed methodology is feasible to expedite oral cancer clinicians in the decision support stage and to better predict the survival rate of the oral cancer patients based on the three markers only.

**Table 11:** Comparison between the current work and the literature

**Author**	**Sample size**	**Accuracy (%)**
Passaro et al. [6]	124 patients, 231 controls	74-79
Oliveira et al. [7]	500	5-year survival of 28.6%
Exarchos et al. [8]	41	100
Exarchos et al. [9]	86	100
Dom et al. [10]	84 patients, 87 controls	82
Current work	31	93.81

A common problem associated with medical dataset is small sample size. It is time consuming and costly to obtain large amount of samples in medical research and the samples are usually inconsistent, incomplete or noisy in nature. The small sample size problem is more visible in the oral cancer research since oral cancer is not one of the top ten most common cancers in Malaysia, hence there are not many cases. For example, in Peninsular Malaysia, there are only 1,921 new oral cancer cases from 2003 to 2005 [39] and 592 new oral cancer cases in the year 2006 [40] as compared to breast cancer, where the incidence between 2003 and 2005 is 12,209 [39] and the incidence for 2006 is 3,591 [40]. Out of these oral cancer cases, some patients are lost to follow-up, some patients seek treatments in other private hospitals and thus, their data are not available for this research. Another reason for small sample size is caused by the medical confidentiality problems. This can be viewed from two aspects, namely, patients and clinicians. Some patients do not wish to reveal any information about their diseases to others, and are not willing to donate their tissues for research/educational purposes. As for clinicians, some may not want to share patients’ data with others especially those from the non-medical fields, while some do not keep their medical records in the correct medical form. From those available cases, some patients’ clinicopathologic data are incomplete, some tissues are missing due to improper management and some are duplicated cases. Due to that, the number of cases that can actually be used for this research is very limited.

In order to overcome the problem of small sample size, we employed the feature selection methods on our dataset to choose the most optimum feature subsets based on the correlations of the input and output variables. The features selected are fed into the proposed classifier and the results showed that the ReliefF-GA-ANFIS prognostic model is suitable for small sample size data with the proposed optimum feature subset of *drink*, *invasion* and *p63*.

### Significance testing

The significance test used in this research was the Kruskal-Wallis test. Kruskal-Wallis is a non-parametric test to compare samples from two or more groups and returns the *p*-value. For this research, we wanted to test if there is any statistical significant difference between the accuracy results generated for the 3-input model of Group 2 for the different feature selection methods. Thus, the null hypothesis is set as: *H*_*0*_ *= There is no difference between the results of the different feature selection models.* If the *p*-value computed from the test is 0.05 or less, the *H*_*0*_ is rejected, which means there is a difference between the results of the different feature selection methods. The *p*-value that generated was 0.0312, which is less than 0.05, this means the *H*_*0*_ is rejected and there is a significant difference between the feature selection methods.

### Validation testing

In this section, the best model of ReliefF-GA-3-input model is compared with other models with a random permutation of three inputs. The purpose is to validate that the features selected by the ReliefF-GA method are the optimum subset for oral cancer prognosis. In addition, the full-input model (the model with all the 17 variables) will be tested as well in order to verify that the reduced model can achieve the same or better results than the full model. In this testing, the classification method used is ANFIS due to its best performance in the previous section and the results are tabulated in Table 12.

**Table 12:** Validation test with random permutation of 3-input model and full input model for Group 2

**Models**	**ANFIS**	
	**%**	**AUC**
**Random permutation model**		
*Age, Inv, p63*	64.76	0.63
*Eth, Dri, p53*	57.14	0.49
*PT, PN, Sta*	58.10	0.51
*Gen, Node, Tre*	70.95	0.59
*Eth, Gen, Sub*	39.05	0.32
*Dri, p53, p63*	80.48	0.70
*Age, p53, p63*	67.14	0.67
*Gen, Dri, Inv*	54.76	0.55
*Site, Inv, Size*	32.86	0.28
*Age, Chew, Size*	48.10	0.41
**Full model**		
Full model with ANFIS	N.A.*	N.A.*
Full model with NN	42.90	0.47
Full model with SVM	54.76	0.46
Full model with LR	54.76	0.59

*N.A. - Results not available due to over-fitting problem as the rule-base generated was too large.

Table 12 presents the results from different permutation of the 3-input models using ANFIS and that of the full model with all the 17 variables using the different classification methods. The three inputs are generated randomly and the best accuracy obtained is 80.48% with an AUC of 0.70. The features selected are *Drink, p53* and *p63.* The results of the full model are not promising and the results of full model using ANFIS cannot be generated due to over-fitting problems as the rule base generated is too large.

Finally, the selected features are tested on the oral cancer dataset for 1-year and 2-year with ANFIS classification and the results are very promising with an accuracy for 1-year prognosis of 93.33% and 2-year prognosis observed at 84.29%, as compared to the 3-year prognosis of 93.81%. The results are shown in Table 13.

**Table 13:** Classification results for 1-year to 3-year oral cancer prognosis

**Oral cancer prognosis**	**Accuracy (%)**	**AUC**
1-year	93.33	0.90
2-year	84.29	0.77
3-year	93.81	0.90

### Findings

The analyses and findings from this research are:

(i) The performance of Group 2 (clinicopathologic and genomic variables) is better than Group 1 (clinicopathologic variables). This is in accordance with the objective of this research, which shows that the prognostic result is more accurate with the combination of clinicopathologic and genomic markers.

(ii) The model with the best accuracy is the ReliefF-GA-3-input model with the ANFIS classification model from Group 2 and the Kruskal-Wallis test showed a significant difference as compared to the 3-input model of GA, CC, ReliefF and CC-GA.

(iii) The optimum subset of features for oral cancer prognosis is *drink, invasion* and *p63* and this finding is in accordance with similar studies in the literature.

(iv) The ANFIS classification model achieved the best accuracy in oral cancer prognosis when compared to artificial neural network, support vector machine and statistical method of logistic regression.

(v) The prognostic result is more accurate with fewer inputs in comparison with the full model.

As a summary, the hybrid model of ReliefF-GA-ANFIS with 3-input features of *drink, invasion* and *p63* achieved the best accuracy. Through the identification of fewer markers for oral cancer prognosis, it is hoped that this will aid clinicians in carrying out prognostic procedures, and thus help them in making a more accurate prognosis in a shorter time at lower costs. Furthermore, the results of this research helps patients and their family plan their future and lifestyle through a more reliable prognosis.

## Conclusions

In this research, we presented a prognostic system using the hybrid of feature selection and machine learning methods for the purpose of oral cancer prognosis based on clinicopathologic and genomic markers. As a conclusion, the hybrid model of ReliefF-GA-ANFIS resulted in the best accuracy (accuracy = 93.81%, AUC = 0.90) with the selected features of *drink*, *invasion* and *p63*. The results proved that the prognosis is more accurate when using the combination of clinicopathologic and genomic markers. However, more tests and experiments needed to be done in order to further verify the results obtained in this research. Our future works include increasing the sample size of the dataset by providing more medical samples thus making it closer to the real population and including more genomic markers in our study.

## Competing interests

The authors declare that they have no competing interests.

## Authors’ contributions

SWC developed the prognostic model, performed the experiments and drafted the manuscript. SWC and SAK conceived with the study and contributed to the experimental design. AFM and RBZ contributed in the analysis and interpretation of oral cancer prognostic dataset. All authors read and approved the final manuscript.
